# Laparoscopic sleeve gastrectomy performed in a morbidly obese patient with gastrointestinal stromal tumor: a case report and literature review

**DOI:** 10.1186/s40792-020-00976-w

**Published:** 2020-08-12

**Authors:** Kenkichi Hashimoto, Yoshihisa Sakaguchi, Sho Nambara, Kensuke Kudou, Eiji Kusumoto, Keiji Yoshinaga, Tetsuya Kusumoto, Koji Ikejiri

**Affiliations:** Department of Gastroenterological Surgery/Clinical Research Institute, National Kyushu Medical Center, 1-8-1 Jigyohama, Chuo-ku, Fukuoka, Japan

**Keywords:** GIST, Morbid obesity, Laparoscopic sleeve gastrectomy

## Abstract

**Background:**

Gastrointestinal stromal tumor (GIST) is the most frequent submucosal tumor, and with advancements of diagnostic modalities, the incidence of GIST cases diagnosed have increased. Similarly, prevalence of morbid obesity has also rapidly increased over the past decade. Notably, the incidence of GIST in obese patients was reported to be more frequent as compared to the general population. Despite local resection being the first choice for GIST treatment, extensive surgery should also be considered depending on the tumor size and location. Laparoscopic sleeve gastrectomy (LSG), the most popular bariatric procedure, could also be a concomitant treatment option for both morbid obesity and GIST when the tumor is contained within LSG the excision range. There are, however, few reports about LSG planned for GIST preoperatively.

**Case presentation:**

A morbidly obese 46-year-old Japanese male (body weight of 105.4 kg, body mass index (BMI) of 36.6 kg/m^2^) was diagnosed with an intramural GIST in the gastric fundus. Because of his extreme visceral fat dominated obesity (visceral fat area of 386 cm^2^), in addition to the size and location of the tumor, we determined that it would be difficult to perform local resection. We planned LSG as a concomitant treatment for both GIST and morbid obesity. After the preoperative examination and 6 months of weight control, the patient lost enough weight to undergo LSG safely. Keeping enough distance away from the tumor, which we observed with an endoscope, we performed LSG to successfully resect the tumor. The patient was discharged uneventfully. Weight loss was successful as his BMI was 21.0 kg/m^2^ at 3 months post-surgery.

**Conclusion:**

We successfully performed LSG in a morbidly obese patient with a large GIST. This is the largest GIST concomitantly resected with LSG reported within current literature.

## Background

Morbid obesity has rapidly increased over the past decade. Bariatric surgery is the most effective therapy to treat obesity and its associated comorbidities. Although laparoscopic sleeve gastrectomy (LSG) is a relatively new bariatric procedure, it overtook the incidence of laparoscopic Roux-Y gastric bypasses globally [1], presumably due to its simple procedure.

Gastrointestinal stromal tumor (GIST) is a very important disease because it is the most frequent and potentially malignant submucosal tumor (SMT). GISTs often originate from the stomach and, as a result of advancements in diagnostic modalities, have recently been detected more frequently. In addition, the incidence of GISTs was reported to be more frequent in obese patients undergoing bariatric surgery (0.8%) as compared to the general population (0.001%) [2]. However, correlation between obesity and GIST is still unknown.

The main treatment for GISTs is complete tumor resection. Local resection is the first choice, but it has been regarded only as the treatment for relatively small or extramural GIST. For large and intramural tumors, extensive surgery such as proximal, distal, and total gastrectomy should be considered depending on the tumor size and location. LSG could also be a concomitant treatment option for both morbid obesity and GIST when the tumor is contained within LSG the excision range. There are, however, few reports about LSG planned for GIST preoperatively.

In this case report, we performed LSG to resect a large GIST located at the gastric fundus in a morbidly obese patient. Here, we report our observation and review the literature on GISTs resected with LSG.

## Case presentation

**Fig. 1:**
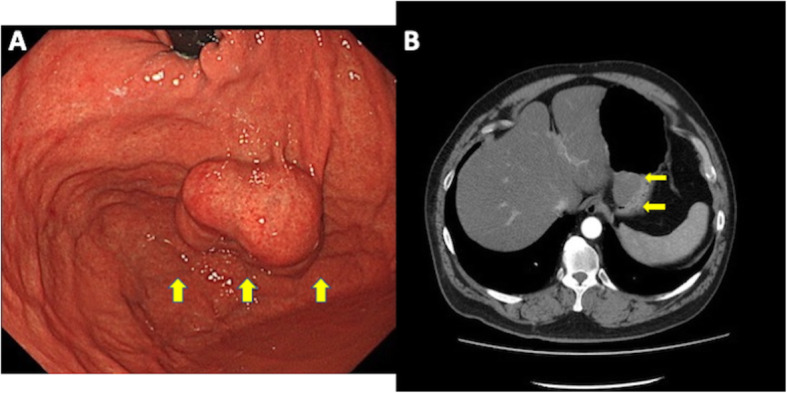
Preoperative imaging found a large SMT (yellow arrows) at the gastric fundus. **a** Endoscopy. **b** CT scan

A morbidly obese 46-year-old Japanese male was diagnosed with a gastric SMT by medical examination and came to our hospital for surgery. The upper gastrointestinal endoscopy and enhanced computed tomography (CT) scan showed an intramural SMT approximately 4 cm in diameter in the gastric fundus (Fig. 1). A biopsy was performed, and histology showed group 1 at that time.

**Fig. 2:**
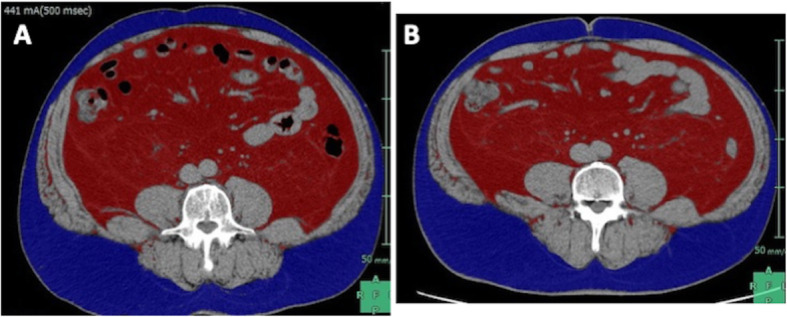
Assessment of abdominal visceral fat (red) and subcutaneous fat (blue) by Fat Scan. The patient lost enough weight, especially visceral fat. **a** Before weight loss. **b** After weight loss

His body weight (BW) was 105.4 kg, and his body mass index (BMI) was 36.6 kg/m^2^. Notably, he had severe visceral adiposity (visceral fat area (VFA) of 386 cm^2^) (Fig. 2a). He also had severe obstructive sleep apnea syndrome (OSAS) (apnea-hypopnea index 79.6) and hypertension. Because of his extreme visceral fat dominated obesity and the size and location of the tumor, we determined that it would be difficult to perform local resection. Instead, we planned LSG as a concomitant treatment for both the tumor and morbid obesity.

After the preoperative examination and 6 months of weight control, the patient lost enough weight (BW 84.2 kg, BMI 29.5 kg/m^2^, VFA 305 cm^2^) to undergo LSG safely (Fig. 2b). In the course of this period, although we checked CT scan every 3 months after first visit, no apparent change was detected. The second upper gastrointestinal endoscopy 6 months after the first one, however, revealed that the tumor had increased in size to approximately 5 cm, and that the tumor was diagnosed as a GIST preoperatively based on the histology results.

**Fig. 3:**
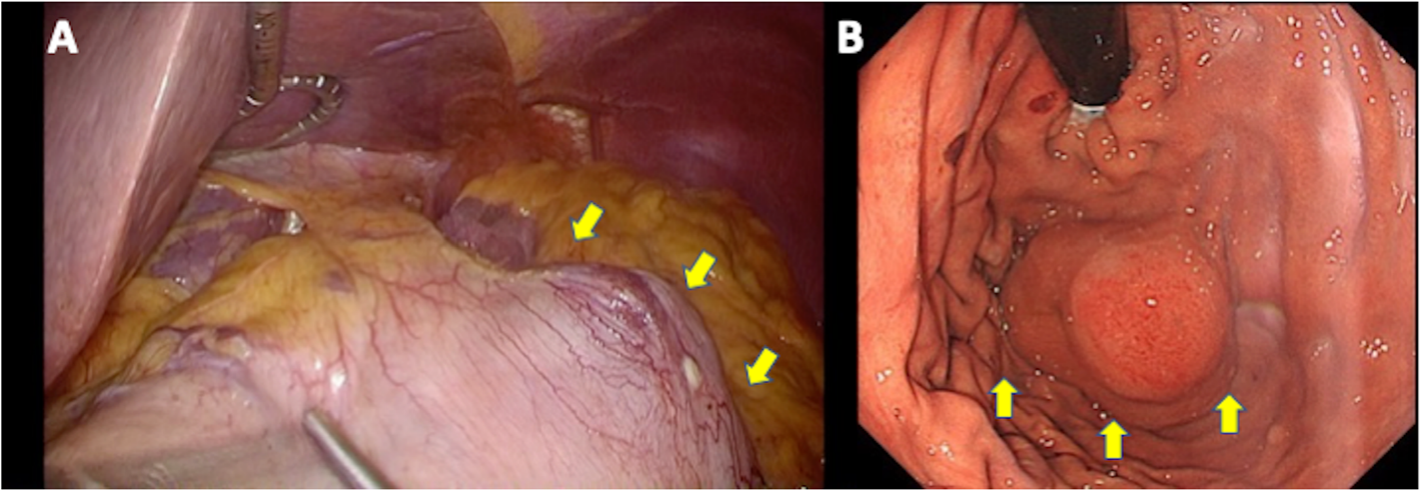
The tumor (yellow arrows) was observed with **a** laparoscopy and **b** endoscopy intraoperatively

We performed LSG in our usual manner except that we advanced endoscopy transorally instead of the 37.5-Fr calibration tube along the lesser curvature to observe the tumor intraoperatively. After complete mobilization of the fundus, we observed the intramural tumor located with enough distance from the esophagogastric junction (EGJ) with both laparoscopy and endoscopy (Fig. 3). The stomach was divided using a linear stapler keeping enough distance from the tumor. We contained the specimen including the tumor in a bag and lengthened the 15-mm port incision site by several millimeters. We could retrieve it as usual manner except above relatively easily. The operation time was 156 min with little blood loss, and the patient was discharged uneventfully on the 7th postoperative day.

**Fig. 4:**
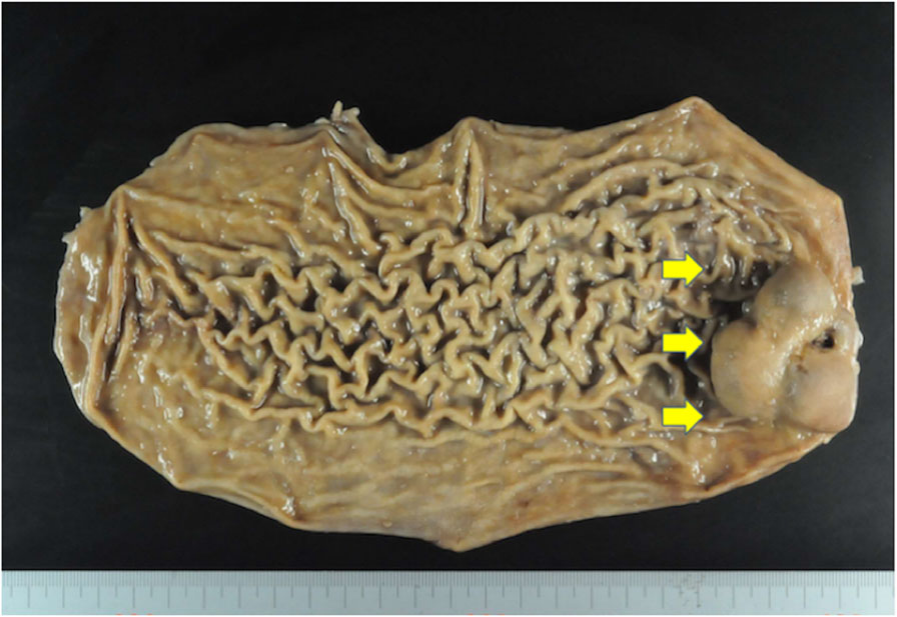
The resected specimen. The tumor (yellow arrows) was resected completely

The tumor, measuring at 6.0 cm × 5.0 cm (Fig. 4), was resected completely and was larger than initial observations during his first visit. Pathological examination revealed that mitotic figures were rarely noted (1/50 HPF), and that the tumor cells were strongly positive to both c-kit and CD34 and negative to S-100, desmin, and alpha-smooth muscle actin immunohistochemically. The GIST was diagnosed as low risk in the Miettinen classification and moderate risk in the Modified-Fletcher classification.

In addition, weight loss was also successful as his BMI was 21.0 kg/m^2^ at 3 months after the surgery.

## Discussion

The clinical practice guidelines for GIST in Japan recommend that the indication for laparoscopic surgery must be decided considering the tumor location including whether intra or extramural, the grade of malignancy of the tumor, and the surgeon’s experience of laparoscopic surgery, when the tumor is over 5 cm in size that are associated with an increased risk of rupture [3]. In this case, because the tumor was intramural and not fragile, we considered that there was not a high risk for rupture. Further, the tumor was located at the gastric fundus, which is the deepest part of the abdomen in a patient with extreme visceral fat obesity. A paper about bariatric surgery mentions that open surgery is known to have high risk for complication, and that laparoscopic surgery is safer and recommended for morbidly obese patients [4]. That is why we judged laparoscopic surgery would be safer than open surgery. Moreover, we have enough experience of laparoscopic surgery including LSG at our institution.

We had four surgical options for this patient: local resection, proximal gastrectomy, total gastrectomy, and sleeve gastrectomy. Although the patient was able to lose weight preoperatively, his BMI and visceral fat area remained high. Considering the extreme visceral fat dominated obesity and the location of the tumor, we determined that local resection would not be impossible but be difficult because of the risk of stenosis at the EGJ. We judged that LSG was easier than local resection because we could cut along the longitudinal axis. Moreover, we judged that LSG was the easiest and safest procedure among the three remaining surgical options because it did not need anastomosis. When a tumor is located at the great curvature of the stomach and contained within the excision range, LSG should be considered the best option. In addition, LSG is a concomitant treatment option for both GIST and morbid obesity. In this case, the patient was considered a high-risk surgical candidate because of his extreme visceral fat dominated obesity and obesity-related comorbidity, such as OSAS. Because medical control is very difficult for morbid obesity patients for a long time, the possibility of weight regain would be very high [5] if we did not perform sleeve gastrectomy. For this reason, we performed LSG as a concomitant treatment for obesity management and resection of GIST. Based on endoscopy and CT scans, we expect successful tumor resection with LSG. However, due to the large tumor size and its location near the EGJ, if we determined LSG resection not to be possible intraoperatively, total gastrectomy would be our second option. Total gastrectomy was simpler than proximal gastrectomy in terms of anastomosis.

The patient underwent weight loss for 6 months, which is an average period in our bariatric program, because of his extreme visceral fat dominated obesity (VFA of 386 cm^2^). Visceral fat and liver volume are known to be the most important risk factors for upper GI surgery including bariatric surgery for morbidly obese patients. In bariatric surgery, we occasionally experience inoperable cases due to significantly enlarged left lobe of the liver and abundant visceral fat disturbing the surgeon’s visualization of upper gastric field. Colles et al. reported that a 5% weight loss leads to an approximately 10% reduction in visceral fat volume and over 20% reduction in liver volume [6]. We judged that preoperative weight loss of at least 10% would reduce the patient’s surgical risk by a sufficient amount even when considering the potential for noncancerous tumor growth while delaying surgical intervention.

The effectiveness of neoadjuvant chemotherapy for GIST has not been established; however, the clinical practice guidelines for GIST in Japan recommend neoadjuvant chemotherapy for selected cases only when the procedure could be diminished [3]. In this case, because the patient was diagnosed with a GIST after the preoperative weight loss period, neoadjuvant chemotherapy was not planned. In addition, if the tumor grew larger or diminished to some extent, we determined that LSG was the best procedure for this case as mentioned above. These were why neoadjuvant chemotherapy was not indicated for this case. As a result, however, the end tumor size during the LSG procedure was larger than anticipated. In retrospect, more frequent checks on the tumor progress should have occurred because GIST is potentially malignant.

A complete literature review was performed ,and reports with reference to GISTs resected with LSG are listed in chronological order (Table 1) [7–22]. Although there have been more than 10 papers on GISTs resected with LSG, most reports described small extramural GISTs encountered during operation and resected incidentally [8–19, 21, 22]. We found only two previous reports on LSG planned preoperatively as the treatment for GISTs that were relatively large and intramural [7, 20]. Wang et al. was the first to report on LSG as a GIST treatment [7]. Chetta et al. reported emergency cases in which LSGs were performed for GISTs with acute bleeding [20]. Notably in our case, the size of the tumor was 6.0 cm × 5.0 cm, which may be the largest GIST resected with LSG planned preoperatively to date.

**Table 1:** A literature review of GISTs resected with LSG

Author	Year	Number of cases	Size of the tumors (cm)	Detection of tumor	Intra/extramural
Wang et al. [7]	2009	2	2–4.4	Pre-op	Intramural
Beltran et al. [8]	2010	1	1.5	Incidental	Extramural
Yuval et al. [9]	2014	5	0.2–2.0	Incidental	Extramural
Chiappetta et al. [10]	2015	7	0.25–1.3	Incidental	Extramural
Crouthamel et al. [11]	2015	12	0.3–2.9	Incidental	Extramural
Ohanessian et al. [12]	2016	3	0.4–0.8	Incidental	Extramural
Atas et al. [13]	2016	1	1.5	Incidental	Extramural
Nickel et al. [14]	2016	1	0.9	Incidental	Extramural
Safaan et al. [15]	2017	11	Not shown	Incidental	Extramural
Viscido et al. [16]	2017	5	0.5–1.5	Incidental	Extramural
Walędziak et al. [17]	2017	14	0.3–2.0	Incidental	Extramural
Saurabh et al. [18]	2017	1	1	Incidental	Extramural
Lyros et al. [19]	2019	4	0.5–1.1	Incidental	Extramural
Chetta et al. [20]	2019	2	4.0–4.5	Pre-op	Intramural
Ayoub et al. [21]	2019	1	2.0	Incidental	Extramural
Mendes et al. [22]	2020	11	0.2–0.9	Incidental	Extramural
Our case	2020	1	6.0	Pre-op	Intramural

## Conclusion

We safely performed LSG in a morbidly obese patient with a large gastric GIST. This is the largest GIST concomitantly resected with LSG among previous reports.

## Data Availability

The datasets generated during the current study are not publicly available because individual privacy could be compromised, but are available from the corresponding author on reasonable request.

## Abbreviations

GIST: Gastrointestinal stromal tumor
LSG: Laparoscopic sleeve gastrectomy
BW: Body weight
BMI: Body mass index
VFA: Visceral fat area
EGJ: Esophagogastric junction
SMT: Submucosal tumor
CT: Computed tomography
OSAS: Obstructive sleep apnea syndrome
